# Correlates of expected eMental Health intervention uptake among Filipino domestic workers in China

**DOI:** 10.1017/gmh.2018.25

**Published:** 2018-10-15

**Authors:** Brian J. Hall, Wei Shi, Melissa R. Garabiles, Edward W. W. Chan

**Affiliations:** 1 Global and Community Mental Health Research Group, Faculty of Social Sciences, The University of Macau, Macau (SAR), People's Republic of China; 2 Department of Health, Behavior and Society, Johns Hopkins Bloomberg School of Public Health, Baltimore, MD, USA; 3 Department of Psychology, Ateneo de Manila University, Philippines

**Keywords:** Domestic workers, eMental Health, migrants, scalable interventions

## Abstract

**Background.:**

Transnational migrant populations face critical barriers to mental health service utilization that perpetuate mental health disparities globally. Overseas Filipino workers (OFWs) number over 2 million globally and 25% are female domestic workers. Structural barriers prevent equitable access to mental health services for this population. Electronic mental health (eMental Health) intervention is a scalable alternative to face-to-face treatment. The current study sought to identify key correlates of intention to use eMental Health within a community of female Filipino domestic workers living and working in Macao (SAR), China.

**Methods.:**

Respondent-driven sampling implemented at a community field site was used to reach a sample of 1364 female domestic workers. A multivariable adjusted partial proportional-odds (PPO) model was used to assess relevant correlates of intent to use eMental Health.

**Results.:**

The majority (62.8%) reported being likely to utilize eMental Health. The adjusted PPO model showed that younger age (18–25, 26–35, 36–45 *v.* over 55), longer time as an OFW, being likely (*v.* neutral and unlikely) to seek professional services, willingness to pay for services (*v.* not), belief that mental health services are a priority (*v.* low priority), having access to Wi-Fi outside the employer's home (*v.* not), and higher levels of social support were associated with increased odds of intent to use eMental Health.

**Conclusions.:**

eMental Health is a promising intervention with high potential for uptake among OFWs. The majority of the study population owned a smartphone and were able to connect to the Internet or Wi-Fi. Future work will rigorously evaluate eMental Health programs for use among OFWs.

## Introduction

Transnational migrant populations face critical barriers to mental health service utilization that perpetuate mental health disparities globally (Zimmerman *et al*. 2011). Providing mental health services to these diverse communities poses a serious global health challenge. Electronic mental health (eMental Health) interventions show promise as efficacious treatments that overcome key barriers to service utilization (Ruzek & Yeager, 2017). At present, limited research assessed the likelihood of vulnerable communities to utilize eMental Health treatment approaches, creating a gap in knowledge for this implementation approach. The present study investigates potential eMental Health uptake among Filipino domestic workers living and working in China.

According to the latest data from the United Nations (2015), the total number of international migrants reached 244 million, and female migrants accounted for more than half of all international migrants (UN, 2015). This figure rose dramatically, by nearly 41%, in the last 15 years. Furthermore, the number of international migrants around the world will surge to 230 million by 2050 (Bhopal, 2007). The Philippines is one of the largest labor-sending countries in the world. There are an estimated 2.2 million overseas Filipino workers (OFW) employed globally, and 25% are employed as domestic workers (Philippine Statistics Authority, 2017). These women enter domestic work despite being educated. Results from a survery of departing domestic workers, 40.3% had high school diplomas and 21.8% had college degrees (Battistella & Asis, 2011).

Previous studies highlighted challenges migrants experience, including poor living and working conditions, long-term acculturative stress, discrimination, physical isolation, loneliness, disruption to family relationships, loss of social status, and disruptions to social networks (Murphy, 1977; Hovey & Magaña, 2002; Bhugra, 2004; Chen *et al*. 2017; Garabiles *et al*. 2017; Hall *et al*. under review). These difficulties help explain why some migrant populations experience a high prevalence of common mental disorders (Bhugra & Jones, 2001; Fassaert *et al*. 2009), suicidal ideation (Ponizovsky & Ritsner, 1999), and experience critical mental health disparities when compared with local populations (Bhugra & Jones, 2001; Pantelidou & Craig, 2006; Lindencrona *et al*. 2008; Fassaert *et al*. 2009; Orozco *et al*. 2013). A systematic review of 35 studies indicated that roughly 20% of labor migrants met criteria for a common mental disorder like anxiety and depression (Lindert *et al*. 2009).

Although a large number of migrants experience mental ill health in their host countries, most will have limited access to mental health services due to various barriers including a lack of available providers who speak their language, stigma and feared potential impact to immigration status, cost of treatment, and difficulties in having sufficient time to seek traditional psychological support (e.g. a psychiatrist or psychologist) (Vega *et al*. 1999; Bhugra & Jones, 2001; Leong & Lau, 2001; Walls *et al*. 2002; Zimmerman *et al*. 2011; Hansen & Cabassa, 2012; Wells *et al*. 2013; Giacco *et al*. 2014; Kaltman *et al*. 2014). If people are not available to deliver interventions who share a similar cultural background, it may also be challenging to seek care due to perceived discrimination from the host culture. Among migrants who live in countries where the mental health care system is not well developed, or insurance is not provided to access these services, mental health treatment will simply not be available (Lindert *et al*. 2009; Eaton *et al*. 2011; Patel *et al*. 2011; Hall *et al*. 2017). In many contexts, low-cost scalable alternatives to in-person psychological treatment by specialist care providers are needed. eMental Health may be a solution (Gaebel *et al*. 2017; Ruzek & Yeager, 2017).

Low-intensity, scalable intervention packages show promise for aiding people with or at risk of a mental illness (Bennett-Levy, Richards & Farrand, 2010; Riper *et al*. 2014; WHO, 2015; Bockting *et al*. 2016). There is growing evidence that eMental Health programs have the potential to optimize and extend traditional mental health treatments, particularly in some low-resource regions where mental health facilities and services are inadequate and limited (Selmi *et al*. 1990; Andersson *et al*. 2005; Wright *et al*. 2005; Titov *et al*. 2010; Wetterlin *et al*. 2014; Arjadi *et al*. 2015).

Additionally, the efficiency of eMental Health has been documented in several systematic reviews (Bee *et al*. 2008; Andrews *et al*. 2010; Riper *et al*. 2011; Andersson *et al*. 2014; Ye *et al*. 2014; Sijbrandij *et al*. 2016). In response to the growing literature supporting the efficacy of eMental Health, the WHO included eMental Health for guided self-help as a psychological treatment in the Mental Health Gap program (WHO, 2016).

The current study had two primary aims. The first aim was to describe the proportion of Filipino domestic workers that would utilize eMental Health. The second aim was to explore correlates of intent to utilize eMental Health, which included participant characteristics, working environment, living environment, previous mental health service use and attitudes about mental health services, stigma, technology use and accessibility, social network support, and current psychological distress. If eMental Health could be successfully implemented among Filipino domestic workers, we would expect that a high proportion of the population would accept this form of intervention and that this intervention would overcome key barriers to traditional psychotherapeutic approaches for migrant communities identified.

## Methods

### Setting and participants

The study was conducted in Macao, China. Macao is a Special Administrative region of China, which is home to 28 377 Filipino Overseas Foreign Workers. Roughly half (13 808) work as migrant domestic workers (Macao Labour Affairs Bureau, 2017). A previous study documented the high burden of mental ill health among the local population and the low number of available mental health providers available (Hall *et al*. 2017). At the time of this study, there are no known mental health providers in Macao that can deliver evidence-based mental health services to migrant workers in either English or Filipino, the official languages of the Philippines.

Data collection was conducted from November 2016 until August 2017 in a field-based setting near the city center and operated by a local non-governmental organization (NGO) that partnered in this research (Macau-Caritas). In total, 1370 Filipino domestic workers were enrolled in the study.

### Study design

Respondent driving sampling (RDS) was used, which is a systematic chain-referral sampling strategy to enroll hard-to-reach populations (Heckathorn, 1997). We selected a total of six ‘seeds’, who were the first people recruited into the study and formed wave 0 (of six referral chains) of the total sample. The seeds were stratified by key population demographic characteristics identified in formative work with the target population (e.g. location, stay-in/stay-out status, age, number of years working as a domestic worker) to maximize the diversity of the subsequent recruitments. They were asked to recruit other individuals in the target population into the study by disseminating recruitment coupons, which were given to them after study briefing and training. Their recruits (wave 1) would come to our study site with the coupons, as a sign of the recruitment chain, and proceeded with the study. Incentives were given to participants for both completing the survey and recruiting their peers using new recruitment coupons provided to them, which created subsequent recruitment referral chains until the target sample size was reached. In total, 1370 domestic workers were enrolled (98.4% from two seeds), and equilibrium was reached by the fourth wave, showing the subsequent sample was independent of bias regardless of any potential bias of the initial selection of the seeds (Heckathorn, 1997).

### Study procedures

Participants entered the research site and informed consent was obtained. After informed consent, all questionnaires were administrated in a quiet and private location using an iPad device in the Filipino language. Study staff, including Filipino research assistants, were available to answer questions if needed during the administration. The Research Ethics Review Committee of the University of Macau approved the study. Following standard RDS procedures, participants received 100MOP (US$12.50) for their participation in the questionnaires. Additional remuneration (20MOP per person; US$2.50) was offered if they successfully recruited up to five additional female Filipino domestic workers who were subsequently enrolled in the study.

### Measures

All measures underwent rigorous translation from English to Filipino. Each item was forward translated, back translated, and underwent cognitive interviewing and pilot testing. One of the authors (BJH), with the assistance of a Filipino research assistant, administered the questionnaires to a small group of Filipino domestic workers. Each item was assessed for understandability and appropriateness for the community in cognitive interviews.

#### Intention to use eMental Health intervention

Participants were asked, ‘If a web-based, or mobile-phone app counselling program was available to you, how likely would you be to use it?’ Responses were unlikely, neutral, and likely.

#### Participant characteristics

Information was gathered about participant age, educational level, marital status, whether they had children, ability to speak and understand Cantonese, number of years as an OFW, number of years working in Macao, and the number of territories and countries worked in, including Macao (1, 2, 3, 4, or more).

#### Working environment

Information was obtained about salary, total weekly working hours, and number of rest days per month.

#### Living environment

Participants indicated if they lived in their employer's home or in a different accommodation (stay-in/stay-out), if they believed they had enough privacy in their living arrangement (0 = no, 1 = yes), and whether they believed their accommodation was safe (0 = no, 1 = yes).

#### Service utilization

##### Past service use

Participants were asked whether in the past 12 months they received any mental health services from the following providers: psychiatrist, psychologist, physician, counselor, social worker, nurse, or NGO worker. Affirmative responses were summed and if the person received any services in the past 12 months, the past service use variable was coded as 1 and no services were coded as 0.

A single variable assessed whether any form of online counseling was used in the past coded 0 = no and 1 = yes.

##### Attitudes and intention about service use

One item was used to assess the likelihood to use professional counseling if it were made available for free (0 = unlikely, 1 = neutral, 2 = likely), how much money they would be willing to pay for treatment (trichotomized into 0 = not willing to pay, 1 = less than 200MOP, 2 = more than 200MOP), and one question asked how important it was to establish mental health services for the Filipino community (0 = low priority, 1 = medium priority, 2 = essential priority).

##### Mental health stigma

Public- and work-related stigma was assessed with two questions related to barriers to past service utilization: ‘People would think I am crazy if I seek help from a professional’, and ‘I was concerned it would affect my job’. Self-stigma was assessed with one question ‘I felt embarrassed or ashamed to seek help’. All three questions were coded 0 = no and 1 = yes.

#### Technology use and accessibility

##### Technology use

We inquired about their frequency of using the following: Facebook or social media, and face-to-face mobile applications to communicate with friends and family members. Responses for each item ranged from 0 = never to 5 = frequently in 1 day.

##### Accessibility

Participants answered whether they owned a smartphone or tablet computer, and whether they could assess the Internet on their smartphone or tablet devices. WI-FI accessibility was assessed from their employer's home, or outside their employer's home, and whether they could maintain an uninterrupted 30-min connection.

*Social network support*. Social support was assessed with the Multidimensional Scale of Perceived Social Support (MSPSS) (Zimet *et al*. 1988), a 10-item scale. Items were summed such that higher scores indicated greater social support. Cronbach's *α* was excellent at 0.97.

#### Current psychological distress

Depression and anxiety symptom severity during the past 2 weeks was assessed using the Filipino versions of the Patient Health Questionnaire-9 (PHQ-9; Kroenke *et al*. 2001) and Generalized Anxiety Disorder-7 (GAD-7; Spitzer *et al*. 2006). These scales demonstrated good reliability and validity in previous studies (Mendoza *et al*. 2017; Mordeno & Hall, 2017). Items for both scales were summed such that higher scores indicated increased symptom severity. Cronbach's *α* for the PHQ-9 was 0.89 and 0.91 for the GAD-7.

### Statistical analyses

A series of univariable χ^2^ analyses tested the association between dichotomous and ordinal correlates and intent to use eMental Health. One-way analyses of variance (ANOVAs) were used to test for the mean between group differences across the intent to use eMental Health outcome for all continuous correlates. ANOVA assumptions were evaluated (STATA sktest command) and correlates that violated assumptions of normality were analyzed using Kruskal–Wallis *H* tests. We followed a purposeful selection of covariates outlined by Hosmer and Lemeshow (2000). In this method, higher *p* value thresholds are set (i.e. 0.10) for univariable analyses in order to include correlates that should be included in final adjusted multivariable models. This approach is known to include covariates that may be missed at the traditional 0.05 level of significance but that are nevertheless important for consideration and inclusion in the model as confounders (Mickey & Greenland, 1989).

Given the ordinal nature of the study outcome, linear regression and logistic regression analyses were not appropriate. A multivariable fully adjusted model including all correlates significant in univariable analyses (with a *p* value set at <0.10) was specified using ordinal logistic regression models (proportional-odds models). The proportional-odds model is a general class of linear model that can deal with ordinal-dependent variables (McCullagh, 1980). Exponentiated model coefficients provide odds ratios that can be interpreted as the log odds of a higher ordinal category (intent to use an eMental Health intervention) *v.* the lower and middle categories (unlikely or neutral intent to use an eMental Health). Ordinal logistic regression assumes proportionality of the odds, which is an assumption often violated in practice. This assumption was tested using likelihood ratio tests and Brant tests (STATA oparallel command). Correlates were shown to violate the proportionality of the odds assumption, and models were respecified using partial proportional-odds (PPO) models (gologit2 command; Williams, 2006), which collapsed the dependent variable into two dichotomous outcomes (not likely = 0 *v.* neutral and likely = 1; and not likely and neutral = 0 *v.* likely = 1). Proportional log odds are interpreted as higher odds of being in the higher category. The PPO model enables parsimonious and flexible modeling of the independent variables (gologit2 auto command option). This model allows variables that violate the parallel assumption to be tested in the same model as variables that do not violate this assumption. The same coefficients are seen across models if they do not violate the parallel assumption. Significance tests for PPO models were two-sided with a critical *p* value set at <0.05. All analyses were conducted in STATA Version 14 (StataCorp, 2015).

## Results

The analytic sample consisted of 1365 female Filipino domestic workers (five participants did not provide data on the outcome). Their mean age was 41.1 (s.d. = 8.9), the majority had children (78.1%), had a college degree (35.9%), were married (44.5%), and worked only in Macao (45.9%). Their median income was 3700MOP (~US$460.00), and the median time working as an OFW was 6 years, with a median 3 years working as a domestic worker in Macao. Their median working time was nearly 68 h per week, with 4 days off per month. The median Cantonese speaking and comprehension was 1 (out of 10). A roughly equal proportion of domestic workers lived with their employers (49.5%) than stayed in separate accommodations. Participant characteristics are displayed in Table 1.

**Table 1.** Participant characteristics

	Median (IQR)	Total *n* (%)
Age group		
18–25		48 (3.46)
25–35		336 (24.2)
35–45		561 (40.4)
45–55		366 (26.4)
55 or older		77 (5.6)
Education		
Elementary		25 (1.8)
High school		504 (36.3)
Technical school		150 (10.8)
Some college		211 (15.2)
College degree or higher		498 (35.9)
Marital status		
Single		352 (25.4)
Married		618 (44.5)
Partnered		99 (7.1)
Separated		225 (16.2)
Widowed		94 (6.8)
Living status		
Stay-in		687 (49.5)
Stay-out		701 (50.5)
Number of countries worked		
1		637 (45.9)
2		428 (30.8)
3		220 (15.9)
4 or more		103 (7.4)
Has children (yes)		1084 (78.1)
Monthly salary (MOP)	3700 (3500–4000)	
Monthly remittance (MOP)	2000 (1000–2500)	
Years as OFW	6 (2–11)	
Years in Macau as DW	3 (1–6)	
Weekly working hours	67.8 (52–78)	
Days off per month	4 (4–4)	
Cantonese proficiency		
Speak Cantonese	1 (0–2)	
Understand Cantonese	1 (0–3)	

A total of 857 (62.8%) of the population reported intent to use eMental Health, compared with 15% who did not intend to use the intervention, and 22% who were neutral. Results from the univariable χ^2^ analyses are presented in Table 2. Kruskal–Wallis *H* tests were conducted for all continuous independent variables since they violated assumptions for normality.

**Table 2.** Results of univariable χ^2^ tests of correlates of intention to use eMental Health

Correlates	Total sample (*N* = 1364) *n* (%)	Unlikely (*n* = 208) *n* (%)	Neutral (*n* = 300) *n* (%)	Likely (*n* = 857) *n* (%)	*p*
*Participant characteristics*					
Age group					0.001
18–25	47 (3.5)	4 (1.9)	12 (4.0)	31 (3.6)	
26–35	334 (24.5)	37 (17.8)	86 (28.7)	211 (24.7)	
36–45	551 (40.4)	76 (36.5)	117 (39.0)	358 (41.8)	
46–55	356 (26.1)	69 (33.2)	75 (25.0)	212 (24.8)	
56 or older	76 (5.6)	22 (10.6)	10 (3.3)	44 (5.1)	
Education					0.79
Elementary	24 (1.8)	4 (1.9)	6 (2.0)	14 (1.6)	
High school	492 (36.1)	83 (39.9)	112 (37.3)	297 (34.7)	
Technical school	146 (10.7)	21 (10.1)	36 (12.0)	89 (10.4)	
Some college	434 (31.8)	65 (34.3)	86 (28.7)	283 (33.1)	
College degree or higher	268 (19.7)	35 (16.8)	60 (22.0)	173 (20.2)	
Marital status					0.09
Married	346 (25.4)	41 (19.7)	76 (25.3)	229 (26.8)	
Single	492 (36.1)	101 (48.6)	140 (46.7)	363 (42.4)	
Partnered	146 (10.7)	16 (7.7)	24 (8.0)	57 (6.7)	
Separated	434 (31.8)	28 (13.5)	44 (14.7)	151 (17.6)	
Widowed	268 (19.7)	35 (16.8)	60 (22.0)	173 (20.2)	
Children					0.77
No	301 (22.1)	43 (20.7)	70 (23.3)	188 (22.0)	
Yes	1063 (77.9)	165 (79.3)	230 (76.7)	668 (78.0)	
*Living environment*					
Stay-in/stay-out					0.01
Live with employer	673 (49.3)	122 (58.7)	140 (46.7)	411 (48.0)	
Live elsewhere	691 (50.7)	86 (41.4)	160 (53.3)	445 (52.0)	
Have enough privacy					0.84
No	260 (19.1)	37 (17.8)	56 (18.7)	167 (19.5)	
Yes	1104 (80.9)	171 (82.2)	224 (81.3)	689 (80.5)	
Feel safe					0.85
No	301 (22.1)	5 (2.4)	8 (2.7)	18 (2.1)	
Yes	1063 (77.9)	203 (97.6)	292 (97.3)	838 (97.9)	
*Past service utilization*					
Past 12 months					0.003
No	1143 (83.7)	191 (91.8)	249 (83.0)	703 (82.0)	
Yes	222 (16.3)	17 (8.2)	51 (17.0)	154 (18.0)	
Past use of eMental Health					0.02
No	1224 (89.7)	196 (94.2)	273 (91.0)	755 (88.1)	
Yes	141 (10.3)	12 (5.8)	27 (9.0)	102 (11.9)	
*Attitudes and intention*					
Intention to use professional services					<0.001
Unlikely	172 (12.6)	82 (39.4)	28 (9.3)	62 (7.2)	
Neutral	321 (23.5)	54 (26.0)	140 (46.7)	127 (14.8)	
Likely	872 (63.9)	72 (34.6)	132 (44.0)	668 (78.0)	
Willing to pay					<0.001
Not willing to pay	237 (17.4)	72 (34.6)	41 (13.7)	124 (14.5)	
Less than 200MOP	677 (49.6)	72 (34.6)	146 (48.7)	459 (55.7)	
More than 200MOP	451 (33.0)	64 (31.8)	113 (37.7)	274 (32.0)	
Attitude toward service needs					<0.001
No or low priority	70 (5.1)	44 (21.2)	10 (3.3)	16 (1.8)	
Medium priority	273 (20.0)	46 (22.1)	104 (34.7)	123 (14.4)	
High priority	1022 (74.9)	118 (56.7)	186 (62.0)	718 (83.8)	
*Stigma*					
Public stigma					0.32
No	1357 (99.4)	207 (99.5)	300 (100)	850 (99.2)	
Yes	8 (0.6)	1 (0.5)	0 (0.00)	7 (0.8)	
Work stigma					0.03
No	1314 (96.3)	203 (97.6)	295 (98.3)	816 (95.2)	
Yes	51 (3.7)	5 (2.4)	5 (1.7)	41 (4.8)	
Self-stigma					0.50
No	1314 (96.3)	202 (97.1)	291 (97.0)	821 (95.8)	
Yes	51 (3.7)	6 (2.9)	9 (3.0)	36 (4.2)	
*Technology use and accessibility*					
Own a smartphone					0.10
No	119 (8.7)	25 (12.0)	29 (9.7)	65 (7.6)	
Yes	1245 (91.3)	183 (88.0)	271 (90.3)	791 (92.4)	
Access Internet on smartphone					0.01
No	101 (22.1)	25 (13.7)	19 (7.0)	57 (7.2)	
Yes	1144 (77.9)	158 (86.3)	252 (93.0)	734 (92.8)	
Own a tablet					0.84
No	1062 (77.9)	165 (79.3)	234 (78.0)	663 (77.5)	
Yes	302 (22.1)	43 (20.7)	66 (22.0)	193 (22.6)	
Access Internet on tablet					0.40
No	10 (3.3)	0 (0.00)	3 (4.5)	7 (3.6)	
Yes	292 (96.7)	43 (100)	63 (95.5)	186 (96.4)	
Access Wi-Fi in employer's home					0.51
No	604 (44.3)	94 (45.2)	124 (41.3)	386 (45.1)	
Yes	760 (55.7)	114 (54.8)	176 (58.7)	470 (54.9)	
Access Wi-Fi outside employer's home					<0.001
No	331 (24.3)	72 (34.6)	79 (26.3)	180 (21.0)	
Yes	1033 (75.7)	136 (65.4)	221 (73.7)	676 (79.0)	
Able to sustain a 30 min Wi-Fi connection					0.08
No	462 (33.9)	84 (40.4)	103 (34.3)	275 (32.1)	
Yes	902 (66.1)	124 (59.6)	197 (65.7)	581 (67.9)	

Fisher's exact test was used in instances where cell sizes were fewer than five.

### Demographic characteristics

The χ^2^ tests and Kruskal–Wallis *H* tests showed that age [χ^2^(8) = 26.092, *p* = 0.001], marital status [χ^2^(8) = 13.633, *p* = 0.09], number of years working as an OFW [χ^2^(2) = 12.373, *p* = 0.0021], and number of countries worked [χ^2^(2) = 10.558, *p* = 0.005] were statistically significantly associated with intent to use eMental Health. Education level, having children, Cantonese language proficiency, and the number of years in Macao were not significantly associated with intent to use eMental Health (*p* > 0.20).

### Working environment

Kruskal–Wallis *H* tests showed that salary, total weekly working hours, and number of rest days per month were not significantly associated with intent to use eMental Health (*p* > 0.50).

### Living environment

The χ^2^ tests showed that stay-in/stay-out status [χ^2^(2) = 8.678, *p* = 0.01] was statistically significantly associated with intent to use eMental Health, whereas safety and privacy of the living environment were not (*p* > 0.80).

### Service utilization and attitudes toward service use

The χ^2^ tests revealed that past 12-month service use [χ^2^(2) = 11.948, *p* = 0.003], past use of eMental Health [χ^2^(2) = 7.530, *p* = 0.02], intention to use professional mental health services [χ^2^(2) = 310.335, *p* *<* 0.001], being willing to pay for services [χ^2^(2) = 56.893, *p* *<* 0.001], and the belief that mental health was important for the community [χ^2^(2) = 195.090, *p* *<* 0.001] were all significantly associated with intent to use eMental Health.

### Stigma

The χ^2^ tests revealed that public stigma in the workplace [χ^2^(2) = 7.215, *p* = 0.02] was significantly associated with intent to use eMental Health, whereas public and self-stigma were not (*p* > 0.32).

### Technology access and use

Kruskal–Wallis *H* tests demonstrated that Facebook and social media use varied by intention to use eMental Health [χ^2^(2) = 16.353, *p* = 0.0003], whereas communication frequency with friends and relatives did not. The χ^2^ tests revealed that owning a smartphone [χ^2^(2) = 4.545, *p* = 0.10], ability to access the Internet on a smartphone [χ^2^(2) = 8.871, *p* = 0.012], ability to access Wi-Fi outside their employer's home [χ^2^(2) = 17.703, *p* *<* 0.001], and the ability to maintain a stable 30-min Internet connection [χ^2^(2) = 5.132, *p* = 0.08] were associated with intent to use eMental Health, whereas owning a tablet computer, access to Internet on a tablet computer, and accessing Wi-Fi in the employers home were not significantly associated with intent to use eMental Health (*p* > 0.50).

### Social support

A Kruskal–Wallis *H* test showed median differences across intent to use eMental Health categories for social support [χ^2^(8) = 39.573, *p* = 0.0001].

### Current psychological distress

Kruskal–Wallis *H* tests demonstrated median differences across intent to use eMental Health categories for depression [χ^2^(2) = 24.711, *p* = 0.0001] and anxiety [χ^2^(8) = 12.525, *p* = 0.002] symptom severity.

### Fully adjusted multivariable PPO model

In fully adjusted multivariable PPO models (see Table 3), younger age groups (18–25, 26–35, 36–45 *v.* over 55), the greater number of years as an OFW, being likely (*v.* neutral and unlikely) to seek professional services, willing to pay (less than 200MOP *v.* not willing to pay), believing that mental health services were important for the community (medium or high priority *v.* low priority), having access to Wi-Fi outside the employer's home (*v.* not), and higher levels of social support were associated with statistically significant increase in the log odds of intent to use eMental Health (*v.* unlikely or neutral).

**Table 3.** Results of adjusted multivariable partial proportional odds models of intention to use eMental Health

	Unlikely *v.* neutral and likely		Unlikely and neutral *v.* likely	
Correlates	Odds ratio (95% CI)	*p*	Odds ratio (95% CI)	*p*
*Participant characteristics*				
Age group				
18–25	2.51 (0.99–6.36)	0.052	2.51 (0.99–6.36)	0.052
26–35	1.93 (1.01–3.68)	0.047	1.93 (1.01–3.68)	0.047
36–45	1.84 (1.02–3.32)	0.044	1.84 (1.02–3.32)	0.044
46–55	1.40 (0.79–2.48)	0.243	1.40 (0.79–2.48)	0.243
56 or older	1.00		1.00	
Marital status				
Married	1.00		1.00	
Single	1.20 (0.87–1.65)	0.270	1.20 (0.87–1.65)	0.270
Partnered	0.72 (0.44–1.17)	0.182	0.72 (0.44–1.17)	0.182
Separated	1.32 (0.93–1.89)	0.123	1.32 (0.93–1.89)	0.123
Widowed	1.14 (0.70–1.87)	0.591	1.14 (0.70–1.87)	0.591
No. of territories worked	1.10 (0.96–1.28)	0.181	1.10 (0.96–1.28)	0.181
No. of years as an OFW	1.00 (0.98–1.03)	0.757	1.04 (1.02–1.06)	0.001
*Living environment*				
Living arrangement (stay-out)	1.19 (0.93–1.53)	0.157	1.19 (0.93–1.53)	0.157
*Past service utilization*				
Service utilization (past 12 months)	1.23 (0.87–1.73)	0.238	1.23 (0.87–1.73)	0.238
Past use of eMental Health	1.46 (0.95–2.24)	0.082	1.46 (0.95–2.24)	0.082
*Attitudes and intention about service use*				
Intention to use professional services				
Unlikely	1.0			
Neutral	3.47 (2.20–5.46)	<0.001	0.97 (0.64–1.46)	0.874
Likely	6.98 (4.58–10.62)	<0.001	4.19 (2.87–6.12)	<0.001
Willing to pay				
Not willing to pay	1.00			
Less than 200MOP	1.65 (1.18–2.29)	0.003	1.65 (1.18–2.29)	0.003
More than 200MOP	1.26 (0.89–1.79)	0.189	1.26 (0.89–1.79)	0.189
Attitude toward service needs				
Low priority	1.0			
Medium priority	4.65 (2.47–8.73)	<0.001	2.60 (1.42–4.75)	0.002
High priority	5.84 (3.35–10.17)	<0.001	5.84 (3.35–10.17)	<0.001
*Stigma*				
Concerned it will affect work (yes)	0.84 (0.31–2.27)	0.732	2.00 (0.92–4.34)	0.079
*Technology use and accessibility*				
Own a smartphone	1.18 (0.78–1.79)	0.435	1.18 (0.78–1.79)	0.435
Frequency of Facebook or social media use	1.17 (1.05–1.32)	0.006	0.97 (0.88–1.07)	0.505
Access Wi-Fi outside employer's home (yes)	1.35 (1.02–1.80)	0.039	1.35 (1.02–1.80)	0.039
Access Wi-Fi sustain a 30 min connection (yes)	1.06 (0.81–1.38)	0.695	1.06 (0.81–1.38)	0.695
*Social resources*				
Social support	1.18 (1.08–1.29)	<0.001	1.18 (1.08–1.29)	<0.001
*Current psychological distress and functioning*				
Depressive symptom severity	1.01 (0.98–1.05)	0.392	1.01 (0.98–1.05)	0.392
Anxiety symptom severity	1.01 (0.97–1.04)	0.668	1.01 (0.97–1.04)	0.668

#### Sensitivity analysis

Live-in employment status was a significant correlate of intent to use eMental Health in the univariable analysis. A higher proportion of domestic workers who live with their employers reported being unlikely to use eMental Health. However, live-in employment status was not significant in the adjusted models. Previous qualitative work revealed that those who stay-in with their employers are more likely to have longer working hours and less freedom more generally. Therefore, as a sensitivity analysis, we re-ran the adjusted PPO model stratified by live-in employment status. No substantive differences in the magnitude of model estimates or significance were observed between groups in this analysis.

## Discussion

This is the first study to ascertain whether transnational Filipino domestic workers would utilize an eMental Health intervention program and assess the correlates that may predict this service use. Over half (62.8%) the sample reported that they would utilize eMental Health and only 15% reported they would not use eMental Health. This figure is suggestive that the majority of Filipino domestic workers in Macao who might need psychological services would use eMental Health if such a program was available.

Older domestic workers would be less likely to utilize eMental Health. In the current study population, and the larger community generally, a small proportion of domestic workers are over age 56. Older adults generally have more difficulty with eMental Health approaches (Keane *et al*. 2013), so additional support or alternative intervention approaches may be more appropriate for this group. A higher proportion of domestic workers reported intent to use eMental Health who worked abroad for longer periods, and in more countries or territories. Years as an OFW remained significant in adjusted models and may be due to the effects of increasing homesickness or cumulative adversities experienced while working aboard (Zimmerman *et al*. 2011).

The working conditions related to salary, working hours, and rest days were not associated with intent to use eMental Health. This is rather counterintuitive given that salary (or cost of treatment) is a traditional barrier to treatment seeking (Kaltman *et al*. 2014; Kantor *et al*. 2017). Moreover, the long working hours and few rest days may interfere with domestic workers ability to engage in eMental Health, but this was not an important barrier in the current analysis.

In Macao, domestic workers may either live within their employer's home, or live in boarding houses (i.e. stay-in/stay-out). Accommodations within the employers’ homes can vary and include private individual rooms, co-sleeping within children or elder's rooms, or sleeping in areas not meant for sleep like in the kitchen or living room. Boarding houses are typically crowded small apartments subdivided with bunk beds that can accommodate up to 10 people. Univariable analysis indicated that residency status was associated with potential eMental Health uptake showing that a larger proportion of domestic workers who lived with their employers reported being unlikely to utilize the intervention compared with those who lived elsewhere. However, residency was non-significant in multivariable analysis, and sensitivity analysis suggested no differences between these groups on key correlates of potential eMental Health uptake.

The majority of domestic workers stated that they found their accommodations both private and safe. Considering that an eMental Health program would likely need to be done outside working hours, it suggests that they could utilize the service from their homes. It is noteworthy that roughly 20% of the sample reported not feeling their accommodation is private, suggesting that problem-solving alternative spaces to use the intervention might be needed so this barrier would be overcome for this sub-group.

Mental health service use in the previous year was reported by 16% of the sample. This is high, and misleading, given the low number of mental health service providers in Macao and the linguistic challenges to deliver care to non-native Chinese communities. For the current analyses, this question was broad, and included all forms of services from numerous sources, including from NGO staff, who are providing largely informal support and organizing activities for migrant workers in Macao. Indeed, disaggregating this variable by service provider demonstrated that 12.9% of those reporting service provision received general support from our local NGO partner organization, Macau-Caritas. Only 3% of the entire sample received any form of specialist or non-specialist mental health services.

This is in stark contrast to the large proportion of the sample reported being willing to seek professional mental health services (>60%), and the majority of the sample reporting being willing to pay for these services. An overwhelming majority of the sample (75%) reported that mental health services were a high priority for the community. This highlights the critical need for mental health provision for this community and supports our previous qualitative study findings that set us on the course of developing these services (Hall *et al*. under review).

In contrast to other Asian populations (Lauber & Rössler, 2007), stigma was notably not high in this study population. The majority of the sample did not report self-, public, or work stigma related to mental health seeking. This suggests that stigma may not be a pervasive issue within the community, or a significant barrier to treatment seeking. Fear of work-related stigma was reported by roughly 4% of the sample. Consequences to working status are especially important for this (and any) transnational migrant community. Interestingly, fear of work stigma was associated with increased intent to use eMental Health in univariable analysis but became non-significant in adjusted models (*p* = 0.07). This pattern suggests that for domestic workers who would not seek services due to fear of work stigma, eMental Health might be seen as a viable solution.

A high proportion (>90%) of domestic workers report owning a smartphone and being able to access the Internet on their smartphone device. A higher proportion of the sample who intend to use eMental Health reported being able to access the Internet on their smartphone. The ubiquity of smartphones and mobile phone data plans suggests a high potential for intervention uptake in the population. Wi-Fi access can also facilitate eMental Health uptake, and a higher proportion of the sample with access to Wi-Fi outside employers’ homes reported intent to use eMental Health. This was also shown in the adjusted model. Consistent with qualitative reports (Hall, Garabiles & Latkin, under review), nearly half the sample could not access Wi-Fi within their employer's home. However, this potential barrier was not associated with intent to use eMental Health in the sample. Moreover, Wi-Fi connections are increasingly made available freely in public parks in Macao, and in most restaurants and cafes, removing any barrier to internet access in this population. Greater use of Facebook and other social media was associated with intent to use eMental Health. This suggests that domestic workers who are more familiar with, or Internet and technology savvy, may be more likely to use eMental Health (Hechanova *et al*. 2013; Price *et al*. 2013).

Social support remained a key correlate of intent to use eMental Health in adjusted models. This could be interpreted to mean that the higher available support, the more willing people would be to seek an intervention. Previous research linked social network support to treatment seeking for mental health (Kantor *et al*. 2017; Vogel *et al*. 2007). It may also be possible that the higher social support available may predict intent to use eMental Health due to the emotional burden that supports network entails (Lincoln, 2000; Newsom *et al*. 2005). Previous studies among Filipino domestic workers noted that social network support (Mendoza *et al*. 2017) and social capital (Hall *et al*. under review) increased psychological distress.

Higher depressive and anxiety symptom severity was associated with intent to use eMental Health in univariable but not adjusted models. Consistent with previous literature, poor mental health alone is not sufficient to seek eMental Health interventions (Hunt & Eisenberg, 2010; McLafferty *et al*. 2017).

The current study is among the few that measured intention to utilize an eMental Health interventions, and to our knowledge, the first to do so among migrant workers. A recent scoping review, which included four studies, reported that intent to use eMental Health interventions was low compared with more traditional, face-to-face treatments (Apolinário-Hagen *et al*. 2017). The review also suggested that people preferred some professional involvement (e.g. guidance) in eMental Health treatment. Although the current study did not compare intent to use eMental Health to other forms of treatment, results showed that participants who expressed intent to use professional services had higher odds of intent to use eMental Health, which is generally consistent with the emerging literature.

### Limitations

The primary limitation of the current study involves the measurement of the study outcome. Although participants all understood the question during cognitive interviews, it remains necessarily generic and does not provide information regarding a specific eMental Health program or its parameters (e.g. number of sessions). This could mean participants would be willing to use such a program in principle but does not provide information about a specific type of eMental Health program that would be best suited for the population. More work will be needed to seek community feedback about the potential uptake of specific intervention programs currently under development. The present analyses did not include other types of OFWs (e.g. engineers, restaurant servers) or men. The generalizability of the findings is restricted to female Filipino Domestic Workers. Some of the contextual variables included in the analysis (e.g. stay-out employment status) do not apply to other countries or territories. For example, labor laws in Hong Kong and elsewhere do not permit domestic workers to live outside their employer's home. The present study is cross-sectional, so causal interpretation of the study results is not possible. The current study did not measure attitudes about expected intervention effectiveness, or preference for eMental Health *v.* other treatment approaches (face-to-face) methods, which can be explored in future research. Finally, the present study is focused on intent to use eMental Health and not on factors that were associated with actual intervention uptake.

Despite these limitations, we believe these data are useful for planning purposes as it allows us to evaluate potential uptake and feasibility for eMental Health intervention in this population. The results point to key strategies that could enhance eMental Health intervention uptake. First, a large proportion of the sample were ambivalent about using eMental Health intervention. Future work is needed to understand how to increase uptake for this group. They may benefit from additional information about eMental Health interventions, their rationale, and how they work. This can be included in promotional and marketing materials (Casey *et al*. 2013). Second, although a large proportion of the population has access to smartphones (>90%), that still may leave 10% of the women without access to the intervention. Providing alternatives (tablet devices, rental phones) to access the program would be useful. Third, and relatedly, Wi-Fi accessibility is a key potential barrier to be overcome to scale this type of intervention, so free or low-cost Internet access may enhance uptake. Fourth, older domestic workers may not be comfortable to use eMental Health interventions. Additional work would be needed to prepare them to utilize the model, or alternative non-technology enhanced interventions might be prioritized for the development of this group. Finally, social support was a correlate of potential uptake, which may indicate utilizing an in-person component to enhance eMental Health uptake.

## Conclusions

The current study suggests that eMental Health approaches are promising for use among a large proportion of female Filipino domestic workers living in Macao (SAR), China. The majority of the study population had a smartphone and were able to connect to the Internet or Wi-Fi. OFWs comprise a population of over 2 million people worldwide and make meaningful contributions to their families through their sacrifices abroad. The burden of mental health is a reality for this population, and scalable evidence-based intervention programs are needed to address this issue. Future work is planned that will culturally adapt and rigorously evaluate the feasibility of eMental Health programs for use among domestic workers and all OFWs.
